# The genomics of poplar-rust interactions to improve tree resistance against fungal disease

**DOI:** 10.1186/1753-6561-5-S7-I12

**Published:** 2011-09-13

**Authors:** Louis-Philippe Hamel, Meriem Benchabane, Ian T Major, Marie-Claude Nicole, Jen Sheen, Armand Séguin

**Affiliations:** 1 Natural Resources Canada, Canadian Forest Service, Laurentian Forestry Centre, Québec, Québec, Canada, G1V 4C7 and Department of Genetics, Harvard Medical School, Boston, Massachusetts 02114, USA; 2 Natural Resources Canada, Canadian Forest Service, Laurentian Forestry Centre, Québec, Québec, Canada, G1V 4C7; 3 1Natural Resources Canada, Canadian Forest Service, Laurentian Forestry Centre, Québec, Québec, Canada, G1V 4C7; 4 Department of Genetics, Harvard Medical School, Boston, Massachusetts 02114, USA

## 

With their long life cycle, trees must have accurate mechanisms to perceive microbial invasion and elaborate signalling networks in order to activate the appropriate defense response through transcriptional reprogramming. Transcriptional activators and repressors participate in the tight regulation of the stress response, which is key to minimise the fitness costs associated with an activated response. With the availability of its whole genome sequence, ease of growth and clonal propagation, and routine transformation, poplar (*Populus* spp.) is considered a model tree species for genomics research and also a good system in forest pathology [1]. Moreover, genomes of a cortege of associated microorganisms are being sequenced including a tree pathogen, *Melampsora* poplar rust. We pursued various approaches to identify poplar genes involved in the interaction with the biotrophic *Melampsora* rust pathogen.

Recent transcriptome analyses from our lab have shown that the expression of genes encoding several transcription factors are up-regulated during infection by *Melampsora* rust. Similarly we have also shown that mitogen activated protein kinases (MAPKs) are associated with poplar disease resistance against *Melampsora* rust [2]. New data obtained from various experimental approaches have directed our focus on two important families of transcription factors; the jasmonate ZIM-domain (JAZ) and Cys2/His2 zinc-finger protein (ZFP) families. We have identified a novel MAPK-interacting partner, *Pti*ZFP1, which belongs to the C2H2 ZFP family of transcriptional EAR repressors.The JAZ family of transcriptional repressors were recently identified as key negative regulators of jasmonate (JA) responses.Transcript analyses show that some ZFP and JAZ members exhibit hormone-related expression profiles and up-regulation by rust infection. This up-regulation of JAZ and ZFP transcripts after rust infection strongly suggests that a hormonal response including JA is a key component of the poplar defence response against *Melampsora*. Late and sustained kinetics of *PtiZFP1* and specific*JAZ* up-regulation suggest that the corresponding proteins may be required for late regulation of defense mechanisms.

Several recent data have shownthat proteolytic cleavage of transcriptional repressors is a general mechanism used by plants to activate gene induction. This mechanism is now well documented for jasmonate signaling, which depends on proteasome-mediated degradation of the JAZ repressors [3,4]. In the present work, we also obtain clear evidence that MAPKs promote degradation of *Pti*ZFP1 through the 26S proteasome.Our work suggests that *Pti*ZFP1 and JAZs are part of *Melampsora* specific hormone-related responses, and by correlation as putative transcriptional repressors, participate in the regulation of the transcriptional responses downstream of these stress hormones.We hypothesize that the observed gene induction play a negative feedback loop needed to replace *Pti*ZFP1 and JAZ proteins that were degraded following through the 26S proteasome. Newly synthesized repressor proteins could contribute to defense attenuation and therefore prevent a runaway response.

Based on our data, we propose a model (Figure 1) where two important families of transcription factors play a predominant role in poplar defense response to the biotrophic fungus *Melampsora*. The activation of poplar MAPKs would lead to*Pti*ZFP1 degradation and as a result to transcriptional activation of defense-related genes. In parallel we also observed that several genes involved in the JA response (including JAZ) are induced following *Melampsora* infection. This model highlights the importance of the 26S proteasome in regulating protein pools of transcriptional repressor. For future studies we intend to uncover which *cis* elements and target genes are recognized by *Pti*ZFP1 and perform functional approaches to uncover JAZ interacting proteins in poplar.

**Figure 1:**
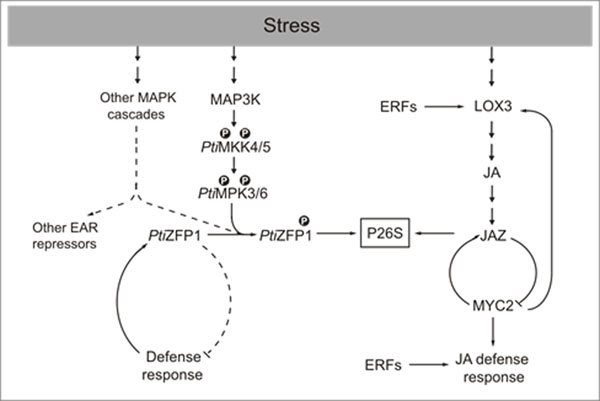
Proposed model of *Pti*ZFP1 function in the plant defense response.

During normal conditions, direct transcriptional repressors such as *Pti*ZFP1 inhibit the expression of stress-related genes. Following stress perception, a MAPK signaling cascade is activated leading to phosphorylation of *Pti*ZFP1. Phosphorylation targets *Pti*ZFP1 for degradation via the 26S proteasome (P26S), thus relieving repression of defense genes. Defense signaling in turn activates *PtiZFP1* gene transcription in order to replenish normal *Pti*ZFP1 protein levels and complete a regulatory cycle necessary to attenuate the defense response. A similar regulatory cycle exists for jasmonic acid (JA) signaling. JA biosynthesis is induced by stress which then promotes proteasome-mediated degradation of JAZ proteins. These indirect transcriptional repressors sequester the bHLH MYC2, thus inhibiting expression of defense genes under non-stressed conditions. Upon release, MYC2 ensures both positive and negative feedback loops by activating *LOX3* and *JAZ* genes respectively. Confirmed and hypothetical pathways are presented in bold and dashed lines respectively.

## References

[B1] Duplessis S Major I Martin F Séguin A Poplar and pathogen interactions: insights from *Populus* genome-wide analyses of resistance and defense gene families and gene expression profiling Crit Rev Plant Sci 2009 28 309 334 10.1080/07352680903241063

[B2] Boyle B Levée V Hamel L-P Nicole M-C Séguin A Molecular and histochemical characterisation of two distinct poplar Melampsora leaf rust pathosystems Plant Biol 2010 12 364 376 10.1111/j.1438-8677.2009.00310.x 20398242

[B3] Chung HS Niu Y Browse J Howe GA Top hits in contemporary JAZ: An update on jasmonate signaling Phytochemistry 2009 70 1547 1559 10.1016/j.phytochem.2009.08.022 19800644 PMC3271379

[B4] Memelink J Regulation of gene expression by jasmonate hormones Phytochemistry 2009 70 1560 1570 10.1016/j.phytochem.2009.09.004 19796781

